# Deployment-related mild traumatic brain injury, mental health problems, and post-concussive symptoms in Canadian armed forces personnel

**DOI:** 10.1186/s12888-014-0325-5

**Published:** 2014-11-20

**Authors:** Bryan G Garber, Corneliu Rusu, Mark A Zamorski

**Affiliations:** Deployment Health Section, Directorate of Mental Health, Canadian Forces Health Services Group Headquarters, 1745 Alta Vista Dr., Ottawa, ON K1A 0 K6 Canada

## Abstract

**Background:**

Up to 20% of US military personnel deployed to Iraq or Afghanistan experience mild traumatic brain injury (mTBI) while deployed; up to one-third will experience persistent post-concussive symptoms (PCS). The objective of this study was to examine the epidemiology of deployment-related mTBI and its relationship to PCS and mental health problems (MHPs) in Canadian Armed Forces (CAF) personnel.

**Methods:**

Participants were 16153 personnel who underwent post-deployment screening (median =136 days after return) following deployment in support of the mission in Afghanistan from 2009 – 2012. The screening questionnaire assessed mTBI and other injuries while deployed, using the Brief Traumatic Brain Injury Screening Tool. Current MHPs and PCS were assessed using items from the Patient Health Questionnaire, the Patient Checklist for PTSD, and the Cognitive Failures Questionnaire. Log-binomial regression explored the association of mTBI, other injuries, and MHPs with PCS, using the presence of 3 or more of 7 PCS as the outcome. Results are expressed as adjusted prevalence ratios (PR).

**Results:**

mTBI while deployed was reported in 843 respondents (5.2%). Less severe forms of mTBI (associated only with having been dazed or confused or having “seen stars”) predominated. Blast was reported as a mechanism of injury in half of those with mTBI. Multiple PCS were present in 21% of those with less severe forms of mTBI and in 27% of those with more severe forms of mTBI (i.e., mTBI associated with loss of consciousness or post-traumatic amnesia). After adjustment for confounding, mTBI had no statistically significant association with PCS relative to non-TBI injury. In contrast, MHPs had a strong association with reporting 3 or more PCS (adjusted prevalence ratio (PR) =7.77).

**Conclusion:**

Deployment-related mTBI prevalence was lower than in many US reports; most of those who had had mTBI were free of multiple PCS. PCS was strongly associated with MHPs but not with mTBI. Careful assessment of MHPs is essential in personnel with a history of combat-related mTBI and PCS.

## Background

Traumatic brain injury is an important public health problem in both civilian and military populations [1]. While brain injury spans the spectrum of mild to severe and can occur in military personnel both on deployment and in garrison, it is deployment-related mild traumatic brain injury (mTBI) that has garnered considerable recent attention [2]. Prevalence estimates for deployment-related mTBI have ranged from 4.4% to 23%, with higher rates being seen in troops with heavy combat exposure [3,4].

In both military personnel and civilians, a variety of acute, transient physical symptoms (e.g., headache, dizziness) and cognitive deficits can result from mTBI [5]. Persistence of those symptoms and/or cognitive impairments after deployment-related mTBI can erode quality of life, interfere with fitness for duty, and serve as a trigger for benefits for veterans [6]. In addition to these concerns about the public health impact of deployment-related mTBI, there has been concern that the pathophysiology and prognosis of blast-related mTBI (a common mechanism in deployment-related mTBI) may differ from that of other injury mechanisms [7,8].

Recovery of acute post-concussive symptoms and neurocognitive deficits occurs in most cases of sports-related mTBI within a short time frame [9]. The persistence of post-concussive symptoms (PCS) beyond 3 months in sports-related mTBI is rare [9], but prevalence rates of persistent PCS have been as high as 15% in other civilian trauma victims [10]. In military personnel with deployment-related mTBI, the prevalence of persistent PCS is at least that high, with post-deployment prevalence rates ranging from 15.8% to 35% [4,11].

The origin of persistent post-concussive symptoms remains poorly understood. Many of these symptoms are common in the general population and are not specific to head injury [12]. However, in those both with and without mTBI, psychosocial factors are clear correlates [13,14]. PCS can overlap with those of post-traumatic stress disorder (PTSD) and depression, both of which can commonly occur in military members following deployment to a combat zone. Indeed, a number of studies of US combat soldiers have shown a strong link of PTSD to persistent PCS [11,15-18]. In addition to the well-documented main effect of mental disorders on PCS, it has also been hypothesized that mental disorders are an effect modifier of the relationship between mTBI and PCS [18].

The bulk of scientific reports on the epidemiology of mTBI incurred during deployment to a combat zone emanate from the United States (US) [11,15-18]. Comparatively, the one other published study done on military personnel from the United Kingdom (UK) showed a much lower prevalence of deployment related mTBI [3]. Differences in deployment experiences such length and nature of exposure in the war zone may also affect prevalence rates, while factors such as cultural differences [19] may impact the expression of PCS. Many of the existing studies also examined the relationship of PCS to PTSD in the post-deployment period [11,15,16], but fewer have explored the relationship to other mental health problems, such as depression and anxiety disorders other than PTSD [3,18]. Disorders other than PTSD also commonly occur in military populations [20], may also be deployment-related [21,22], may be consequences of brain trauma [23], and may also have symptom overlap with PCS [24].

Hence, the fundamental purpose of this study is to examine the epidemiology of mTBI in a different military population using data from a large population-based clinical screening program that employs the same case definitions and screening tools as most of the published US studies. Specifically, it uses data collected during the mandatory Enhanced Post-deployment Screening process on Canadian military personnel deployed in support of the mission in Afghanistan to:

1. Determine the prevalence of self-reported mTBI while deployed;
2. Determine the prevalence of multiple PCS in the post-deployment period in those with and without a history of deployment-related mTBI;
3. Explore the role of a broader range of mental health problems and mTBI with PCS in the post-deployment period; and

## Methods

### Respondents

Respondents were 16193 CAF personnel who deployed in support of the mission in Afghanistan who also completed a version of the Enhanced Post-deployment Screening (EPDS) questionnaire with questions on mTBI over the period January 2009 – July 2012. These individuals had deployed largely for six to eight months to Kandahar Province (Afghanistan) or in the Persian Gulf region, and they had fulfilled a broad range of combat, peacekeeping, operational support, administrative, and other roles.

### The EPDS Process

CAF policy requires that personnel deployed for 60 days or more undergo EPDS between 90 and 180 days after return to Canada. The median period of completion for the period of this study was 136 days after return from deployment (interquartile range: 100–178 days). EPDS consists of completion of a detailed health questionnaire followed by an in-depth interview with a mental health professional. At least 76% of those who require screening under the policy actually complete it [25].

### Screening questionnaire content

#### Mild traumatic brain injury

mTBI was assessed using the first two questions of the Brief Traumatic Brain Injury Screening Tool, which was developed in a deployed US military population [26]. The first item asks whether the respondent was injured during the deployment from any of the following mechanisms: fragment, bullet, vehicular, fall or blast. The second item assesses symptoms of alteration in mental status *immediately* after the injury. The screen is considered positive in those who acknowledge an injury associated with being dazed, confused, or seeing stars, having loss of consciousness, or having post-traumatic amnesia at the time of the injury or immediately thereafter. This criterion has been shown to have a sensitivity of 80% and specificity of 93% for clinician diagnosed deployment related mTBI [27]. To keep the focus of the study on *mild* TBI, subjects reporting loss of consciousness of greater than 20 minutes (N =40) were excluded, leaving 16153 in the final data set.

#### Common mental health problems

In order to facilitate the exploration of the relationships among mTBI, PCS, and MHP’s, we assessed MHP’s using instruments that did not include any symptoms that overlap with those defined in the ICD-10 definition of PCS, such as feeling tired, trouble concentrating, sleep problems, and irritability. Depression was assessed with the 2-item Patient Health Questionnaire [28] (PHQ-2)(recall period =2 weeks; response categories = “not at all,” “several days,” “more than half the days,” and “nearly every day”; range 0 to 6; Cronbach’s α =0.80) with a cut-off point of 4 or greater [29]. This cut-off has 73% sensitivity and 93% specificity for a clinical diagnosis of major depressive disorder in primary care patients [29]. Post-traumatic stress disorder (PTSD) was assessed using the 2-item [30] PTSD Checklist – Civilian Version [31] (PCL-2, recall period =30 days; response categories = “not at all,” “a little,” “moderately,” “quite a bit,” and “extremely”; range 2 to 10; α =0.81), with a cut-off of 6 points or greater. This cut-off was chosen using the study data to minimize misclassification relative to the use of the full PCL scale with a conventional diagnostic cut-off of 50 or higher [31]. A cut-off of 6 or greater on the PCL-2 was associated with a sensitivity of 89%, a specificity of 97%, and 99% agreement with a full PCL score of 50 or greater. Panic disorder was assessed using the PHQ, with a modified algorithm that did not require the presence of four or more symptoms during the most recent panic attack [32] (4 items; recall period =4 weeks; response categories = “no,” and “yes”; α =0.89). The aggregate outcome of “any mental health problem” included those that reported symptoms consistent with one or more of the foregoing.

#### Post-concussive symptoms

Seven symptoms were assessed using items that preceded the mTBI screen; no explicit linkage with TBI was made on the questionnaire. Headache and dizziness, were assessed using individual items from the PHQ (recall period =2 weeks; response categories = “not at all,” “bothered a little,” and “bothered a lot”); fatigue and difficulty concentrating were assessed using two items from the PHQ depression scale; insomnia and irritability were assessed using the corresponding items from the PCL-C. Memory problems were assessed using an item adapted from the Cognitive Failures Questionnaire, using a recall period of two weeks and response categories of “not at all,” “bothered a little,” and “bothered a lot.” [33].

#### Combat exposure

A modified, 30-item version of the scale developed by Walter Reed Army Institute for Research (US) was used to measure the extent of combat exposure [34]. Each item was a simple yes/no question regarding having experienced specific traumatic experiences while deployed, and the scale score was simply the sum of positive responses (range 0 to 30, α =0.91). For analysis purposes, the scale score was divided into tertiles, determined with respect to a larger reference population of Canadian Armed Forces personnel undergoing post-deployment screening after a number of different military operations since 2009.

#### Sociodemographic and military characteristics

These potential confounders were assessed using items developed for the EPDS questionnaire: Sex, age, language, marital status, rank, component (Regular vs. Reserve Force), element (Army, Navy, or Air Force), years of military service, number of previous deployments, deployment length, and timing of screening relative to return from deployment. Missing data were filled in where possible using administrative data sources.

### Primary outcomes

The primary outcomes were:

1. *Self-reported mTBI while deployed.* mTBI was stratified using the immediate symptoms after the injury, with injuries associated with only being dazed, being confused, or seeing stars being considered less severe than those associated with loss of consciousness (LOC) or post-traumatic amnesia (PTA) being considered more severe [35]. We modeled injury status as a four-level variable as originally described by Hoge et al in their seminal paper on mTBI following deployment to Iraq: [18] Uninjured, injured without mTBI, less severe mTBI, and more severe mTBI. There were several reasons for adopting this approach. First, some have questioned the validity of the criteria of dazed or confused only in establishing mTBI in a combat setting, arguing that it may be a manifestation of acute combat stress reaction and not related to head injury at all [24]. Secondly, studies in a civilian setting have found that the symptoms of PCS are similarly prevalent in injured persons with and without a history of head injury [12]. Including a spectrum of injury and mTBI severity allowed a more complete assessment of the specificity of PCS in a military population.
2. *Current post-concussive symptoms.* The definition of post-concussive symptoms used in this analysis is modelled after the World Health Organization ICD-10 definition for post-concussion syndrome. Our operational definition was modeled after those used in previous US military studies exploring the relationship between mTBI, post-concussive symptoms and mental health problems [4,11]. Specifically, those with three or more of the following seven symptoms were considered to be post-concussive symptom cases: (headache, dizziness, memory problems, fatigue, difficulty concentrating, insomnia and irritability—see above for source of items. The recall period for the symptom items ranged from two weeks to 30 days, in accordance with recall period used in the parent scale (see above). Only those with more intense or frequent symptoms were considered to be positive: “bothered a lot” [headache, dizziness, memory problems], “bothered more than half the days” or “nearly every day” [fatigue, difficulty concentrating], or “bothered moderately” or greater [insomnia, irritability]. The reliability of these seven items (each treated dichotomously as described above) was α =0.70. The ICD-10 definition explicitly includes anxiety and depression as symptoms of post-concussion syndrome, but these were not included in our definition because doing so would have made it impossible to explore the independent role of mental health problems and TBI in the genesis of PCS. Finally, given that these symptoms were assessed 3 to 6 months post-deployment, and that we did not definitively ascertain the time course of symptoms, PCS is described as “current.”

### Statistical analysis

To simplify analysis and interpretation, for individuals who completed more than one EPDS questionnaire over the study period (N =972), only the first EPDS questionnaire was used in this analysis. Because this data represents a near census of the recently deployed population, confidence intervals for prevalence rates are not reported. The covariates hypothesized to confound the relationship between injury status and reporting three or more PCS were identified *a priori* based on literature review. As the outcome was not rare, a multivariate log-binomial regression model was used to estimate the crude and adjusted prevalence ratios (PRs) for the association between injury status and reporting three of more PCS. Combat exposure, age, sex, language, component, element, and rank were identified as potential confounders. In order to be consistent with previous studies [3,11,18,36], the injured without mTBI group served as the reference category in all the regression analyses, and uninjured participants were excluded.

Listwise deletion resulted in the exclusion of 197 cases (5.5%) from the regression analysis, leaving 3351 in the final regression model. Analysis was done using SAS for Windows, version 9.3.

### Ethical aspects

The study protocol was approved by Veritas Research Ethics Board (Montreal, Canada) and the Directorate of Access to Information and Privacy (DAIP) of the Department of National Defence. DAIP determined that the proposed use of existing administrative and health data was consistent with Paragraph 8(2)(j) of the *Privacy Act* which allows the head of the institution to permit disclosure of personal information for research activities. Consequently, written informed consent was not required.

## Results

As shown in Table 1, respondents were largely male, junior non-commissioned members in the Regular Force with substantial military experience. A broad range of combat exposure was seen (median =6 exposures, interquartile range =3 to 12). mTBI while deployed was reported by 843 respondents (5.2%), with most of these (61.8%) in the less severe mTBI group. Almost half of mTBI cases (48.8%) were blast-related. An additional 16.8% reported non-TBI injuries. Combat exposure increased the risk of mTBI, with 12.61 (%) of those in the highest tertile reporting mTBI vs. 1.15 (%) in the lowest tertile. 1356 (8.8%) had symptoms of one or more of the three mental health problems assessed, with the prevalence of PTSD, depression, and panic disorder being 5.7%, 3.7%, and 1.9%, respectively. Three or more PCS were reported by 1401 respondents (8.7%).

**Table 1:** **Military and socio-demographic characteristics (Overall N =16153)**

		**N**	**%**
Age	24 years or less	3134	19.42
	25 – 34 years	7075	43.84
	35–44 years	4049	25.09
	45 or more	1882	11.66
	Total	16140	
Sex	Male	14641	90.67
	Female	1507	9.33
	Total	16148	
Rank	Junior NCM*	10940	67.81
	Senior NCM^†^	2863	17.75
	Officer	2331	14.45
	Total	16134	
Component	Regular	13812	85.52
	Reserve	2339	14.48
	Total	16151	
Element	Land	12824	79.54
	Sea	959	5.95
	Air	2340	14.51
	Total	16123	
Language	English	11828	73.50
	French	4185	26.00
	Other	79	0.50
	Total	16092	
Years of military service	5 or less	5236	32.42
	6 to 15	6240	38.64
	16 or more	4675	28.95
	Total	16151	
Number previous deployments	0	7516	46.96
	1–2	5025	31.40
	3 or more	3464	21.64
	Total	16005	
Combat exposure (tertiles)^‡^	1st Tertile	4535	28.71
	2nd Tertile	6397	40.08
	3rd Tertile	5029	31.51
		15961	
Injury status	Uninjured	12602	78.02
	Non-TBI injury	2708	16.76
	Less severe mTBI	521	3.23
	More severe mTBI	322	1.99
	Total	16153	
Major depression	No	15560	96.33
	Yes	593	3.67
	Total	16153	
PTSD	No	15233	94.30
	Yes	920	5.70
	Total	16153	
Panic disorder	No	14747	98.06
	Yes	291	1.94
	Total	15038	
Any MHP	No	14058	91.20
	Yes	1356	8.80
	Total	15414	

*Junior non-commissioned member (NCM) includes rank of Master Corporal (or equivalent) or below.

^†^Senior non-commissioned member (NCM) includes rank of Sergeant (or equivalent) or above.

^‡^Tertiles are computed relative to a larger reference population, which accounts for the uneven distribution of cases. The cut-off used were: 0–2; 3–10; 11 + .

As shown in Table 2, three or more PCS was reported by both mTBI groups (27.0% and 21.4% for the more and less severe mTBI groups, respectively). However, those with non-mTBI injuries also had a substantial prevalence of PCS (13.3%) relative to the uninjured (6.7%). Indeed, 1203 out of 1401 respondents (86%) with PCS were in the group *without* mTBI. Current PCS was far more prevalent in those with any mental health problems compared to those without (55.2% vs 3.8%).

**Table 2:** **Prevalence of reporting three or more PCS, stratified by injury status and MHP**

	**No MHP**		**MHP**		**Total**	
	N	**%**	**N**	**%**	**N**	**%**
Injury status						
Uninjured	344	3.06	426	54.06	846	6.73
Non-TBI injury	148	6.62	185	56.40	357	13.25
Less severe mTBI	24	6.82	73	53.68	111	21.35
More severe mTBI	20	9.71	60	63.16	87	27.02
Total	536	3.82	744	55.23	1401	8.69

Table 3 shows the association between injury status and reporting three or more PCS, with adjustment for MHP, age, sex, language, component, element, rank, and combat exposure. With the exception of MHP, sex and rank, none of the covariates were associated with reporting 3 or more PCS in the adjusted model. Injury status had no statistically significant association with PCS. Indeed, when compared with those with non-TBI injuries, the group with less severe mTBI had no elevated risk for PCS (adjusted PR =0.97; 95% CI:0.82-1.15), while the group with more severe mTBI was only marginally associated with PCS (adjusted PR =1.16; 95% CI:0.98-1.38). In contrast, MHP had a strong association with reporting 3 or more PCS (adjusted PR =7.77; 95% CI:6.60-9.15). Higher risk for PCS was also observed for female sex (adjusted PR = 1.34; 95% CI:1.10-1.63). Being an officer was associated with lower risk for PCS (adjusted PR = 0.76; 95% CI;0.50-0.98). There was no evidence of an interaction effect between MHP and injury status in the expression of PCS in the regression model using the non-mTBI injury group as the reference category (results not shown).

**Table 3:** **Unadjusted and adjusted PRs of reporting three or more PCS (N = 3351)**

**Covariate**		**Unadjusted PR (95% confidence interval)**	Adjusted* PR (95% confidence interval)
Injury status	Non-TBI Injury	1.00	1.00
	Less severe mTBI^†^	1.61(1.33-1.95) p < 0.0001	0.97(0.82-1.15)
	More severe mTBI^†^	2.04(1.66-2.50) p < 0.0001	1.16(0.98-1.38)
Combat exposure^¶^	1st Tertile	1.00	1.00
	2nd Tertile	1.16(0.87-1.55)	0.94(0.73-1.22)
	3rd Tertile	1.82(1.40-2.37) p < 0.0001	1.07(0.83-1.38)
Age	24 years or less	1.00	1.00
	25 – 34 years	1.22(0.98-1.52)	1.19(0.98-1.43)
	35–44 years	1.20(0.94-1.52)	1.21(0.97-1.51)
	45 or more	1.05(0.78-1.42)	1.42(0.86-1.22)
Sex	Male	1.00	1.00
	Female	1.50(1.16-1.93) p = 0.002	1.34(1.10-1.63) p = 0.004
Language	English	1.00	1.00
	Non-English	0.82(0.67-1.00) p = 0.049	1.02(0.86-1.22)
Element	Land	1.00	1.00
	Sea	0.69(0.45-1.04)	0.79(0.53-1.17)
	Air	0.75(0.57-0.99) p = 0.04	0.87(0.67-1.11)
Rank	Junior NCM^‡^	1.00	1.00
	Senior NCM^§^	1.05(0.87-1.27)	0.96(0.81-1.14)
	Officer^¶^	0.52(0.37-0.74) p = 0.0003	0.76(0.50-0.98)
Any MHP	No	1.00	1.00
	Yes	8.28(7.10-9.66) p < 0.0001	7.77(6.60-9.15) p < 0.0001

*Adjusted for injury status, combat exposure, age, sex, language, component, element, rank and MHP.

^†^More and less severe mTBI included those with and without loss of consciousness or post-traumatic amnesia, respectively.

^‡^Junior non-commissioned member (NCM) includes rank of Master Corporal (or equivalent) or below.

^§^Senior non-commissioned member (NCM) includes rank of Sergeant (or equivalent) or above.

^¶^Tertiles are computed relative to a larger reference population, which accounts for the uneven distribution of cases.

## Discussion

### Key findings

Deployment-related mTBI was reported in (5.2%) of Canadian Armed Forces members deployed in support of the mission in Afghanistan. Three-quarters of those with mTBI were free of PCS when screened after their return. A history of mTBI, whether manifested as altered consciousness or loss of consciousness, failed to show a significant independent association with PCS after controlling for other confounders. In contrast, the presence of MHP’s showed a powerful association with the presence of PCS in this military cohort.

### Comparisons with the findings of others

#### Prevalence of deployment-related mTBI

Our prevalence rate (5.2%) is lower than that reported in many US reports (range from 12-23%) [4,11] and more similar to that reported by the UK (4.4%) [3]. Such differences may be accounted for by differences in deployment duration and the degree of combat exposure. Indeed, a subsequent analysis on mTBI incidence rates using the same UK data set showed that the differences between the UK and US estimates began to narrow when accounting for the period of exposure (i.e., the deployment duration) [37]. Our finding showing a clear relationship between combat exposure and prevalence of self-reported mTBI is consistent with prior observations [3,18]. Unfortunately, none of the existing publications on military mTBI report combat exposure in a similar enough manner to allow a determination as to whether this may contribute to differences in prevalence estimates. Ultimately, there is no single prevalence estimate of mTBI in a deployed military population. This highlights the need for all militaries to estimate the risk based on the nature and duration of the deployment.

#### Prevalence of PCS in those with mTBI

This study found that current PCS were seen during post-deployment screening in 27.0% and 21.3% of those with more and less severe mTBI, respectively. US military studies using a similar case definition have noted similar rates (15 – 35%) [4,11]. Estimates of PCS in the civilian trauma victims (10-15%) [10] lie between the rates seen in military populations but likely above those with sports-related concussion [9]. Differences in the underlying prevalence of mental health problems and the context in which the mTBI occurred may account for the differences in PCS prevalence seen in different populations.

#### Lack of specificity of PCS

Our finding of the association of PCS with non-TBI injury is consistent with both military and civilian studies showing a similar lack of specificity of PCS [12,18]. Indeed, many researchers have questioned the validity of PCS as a true syndrome or disease entity [10].

#### Association of mental health problems, mTBI, and PCS

The extremely strong association between mental health problems and PCS has been noted by many other civilian [39-41] and military studies [11,15-18,41-45]. Although much of the military literature has primarily focussed on PTSD [11,15,16,20,44], a few have examined the relationship between other mental health problems, mTBI and PCS. Hoge et al found that the association of mTBI with post-concussive symptoms other than headache were no longer significant after adjustment for PTSD and depression [18]. A more recent report from a deployed UK military population found that only 3 of 9 PCS symptoms remained associated with mTBI after adjustment for current PTSD symptoms as well as psychological distress and alcohol misuse recorded before deployment.

### Limitations

The cross-sectional nature of this study means that the observed associations may not be causal. In particular, it is possible that mental disorders (especially depression) may lie along the causal chain between injury and PCS [46]. Underreporting of mental health problems is also likely [47-49], given the non-anonymous nature of the EPDS questionnaire. However, studies using anonymous data have yielded comparable findings with respect to the associations among PCS, mental health problems, and mTBI [18].

History of mTBI was obtained through self-reports so recall bias is possible. More importantly, 62% of our TBI cases reported being dazed/confused or seeing stars as their sole symptom. Current military case definitions for mTBI include this group [50,51], but interpreting these symptoms is difficult when head trauma coincides with psychological trauma in a combat setting, and particularly when blast is a primary injury [24]. Dissociative symptoms are common after severe trauma, and distinguishing these from alteration of conscious in the aftermath of events that are both physically and psychologically traumatic can be challenging. This raises the possibility that some cases who report being dazed/confused only are misdiagnosed as mTBI when they may in fact be attributable to acute psychological trauma. However, this study found that the strength of the association between mTBI and PCS was similar in those with LOC or PTA as well as the less severe mTBI group that reported being dazed and confused only. Nevertheless, reliance on clinical criteria alone to diagnose mTBI has limitations [24,52]. The absence of a reliable and validated objective measure of mTBI will continue to hamper our efforts to understand the natural history of this injury and disentangle its clinical course from that of co-morbid psychological problems.

Universally accepted research criteria for establishing post-concussive syndrome do not currently exist [53], and there is emerging evidence that some such symptoms are more specific than others [3,18]. Nevertheless, all formal case definitions have at their core the concept of multiple symptoms of the sort we included in our case definition.

The definition employed for post-concussive syndrome in this paper was based on ICD-10 and has been used in other military studies [4,11] but civilian research has shown that this criterion is less specific than others [54-56]. This is particularly salient when looking for symptoms of mental health problems in those with PCS given the overlap of these symptoms between the two conditions. We strove to mitigate that potential effect by excluding anxiety and depression (present in the ICD-10 criteria) from our operational definition of PCS. Furthermore, depression and PTSD were evaluated using PHQ-2 and PCL-2 scales that excluded the symptoms considered for the assessment of PCS. We were unable to apply the full symptom criterion for PCS in the ICD-10 definition (intolerance of stress, emotion, or alcohol) because we did not have any items that mapped neatly to that construct, meaning that our threshold for PCS (3 of 7 symptoms vs. 3 of 8 symptoms specified by ICD-10) is more stringent than the ICD-10 definition. The primary advantage of our approach is that it enhanced comparability with some international findings. Until a better universally adopted definition of PCS is established and reliable cut-offs for a minimum number of PCS that reliably distinguish between symptoms due to mTBI as opposed to other causes, this problem will continue to hamper our understanding of these phenomena.

Although debate continues about the long term sequelae of repeated head injuries [57-60] we were not able to assess whether a history of repeated concussion would have explained part of the variation observed in the expression of PCS in this population. Similarly, we did not explore whether any of the study subjects were seeking financial compensation for injuries, a factor which is known to affect the manifestation of PCS [61]. It is therefore possible that over-reporting of mTBI history and persistent PCS may have occurred in those desiring possible benefits and compensation. Finally, we were not able to account for pre-deployment health or other characteristics (e.g., personality, past history of mental health problems, or past concussions) in our analysis.

This study does, however, have some key strengths: By adding data on another non-US population using comparable assessment tools and case definitions, it furthers our understanding of factors which may influence frequency estimates of mTBI in different military populations. Our large sample size permitted controlling for a number of covariates in assessing the relationship between deployment-related mTBI, PCS and mental health problems. Specifically, it allowed an evaluation of a broader range of potential mental health problems beyond the more typically studied PTSD and afforded the ability to detect relatively small effects.

### Implications

Those who report multiple symptoms many months following a history of deployment-related mTBI present a complex clinical picture. Psychological illnesses such as PTSD, depression and substance abuse [22,62-64], are prevalent following military deployment and are associated with somatic symptoms in and of themselves [65]. Others experience medically unexplained physical symptoms not accounted for by common mood, anxiety, or substance use disorders, an observation that initially emanated from the 1990 – 1991 Persian Gulf War but is now largely recognized to have existed in historical conflicts as well [66].

The results of this study add to an existing body of literature on factors correlated with the expression of PCS by demonstrating that mental health problems are the strongest factor associated with PCS in individuals with a deployment related mTBI. The question as to why this association exists remains a matter of conjecture. A recent review on the etiology of persistent PCS examined the plausibility of several explanations [67]. One of these is that the association is an artefact of symptom overlap between PCS and comorbid psychiatric conditions [38]. While this likely is a contributing factor to effect sizes observed in the literature, our study still observed a strong association of mental health problems with PCS even after removing the most highly overlapping mental health symptoms (depression, anxiety) in our PCS definition. Similarly, another study of military veterans found that in-theatre PTSD was more associated with health outcomes then mTBI even after excluding irritability and difficulty concentrating from their PTSD measure [16]. Another hypothesis is that mTBI acts as a “third common-causal variable” by producing both PCS and psychiatric disorders [67]. Theoretically, neurochemical changes that develop in the acutely injured brain could contribute to the development of later psychiatric pathology in the form of depression, PTSD, and generalized anxiety disorder. However, it is unclear whether the development of a psychiatric syndrome is the pathophysiologic consequence of brain injury or is a manifestation of a generalized psychological response to trauma and/or the resulting disability [68]. Although each of these explanations may account for some of the variation in the expression of persistent PCS, the likely stronger explanation is that psychological factors play an important role in the earliest stages of recovery from mTBI [67]. This is based on evidence that psychological distress is fairly common in the initial days after an mTBI and correlates with initial PCS severity [69,70] and is among the best acute stage predictors of late PCS outcome [13].

Regardless of the causal pathway between PCS and mental health problems, the implication of this finding for military health care providers is that careful assessment of the full spectrum of common mood, anxiety, and trauma-related disorders must be an integral component of a comprehensive medical evaluation of military personnel who have persistent PCS.

Future research on deployment-related mTBI needs to better address the problem of misclassification of mTBI cases that inevitably results when it co-occurs with psychological trauma. Studies using case-definitions that rely on objective pathophysiological findings, such as biomarkers or neuroimaging, are desperately needed. Similarly, there is a need for more longitudinal studies having adequate, unbiased information about pre-injury characteristics such as personality factors and prior mental health problems. This would allow better identification of populations at risk of persistent PCS and improved targeting of early intervention strategies, if any are eventually developed [71]. Finally, further evaluation of the specificity of particular post-concussive symptoms (e.g., headache) is warranted in order to develop a more specific case definition for post-concussion syndrome.

## Conclusion

The prevalence of deployment-related mTBI and PCS in CAF personnel deployed in support of the mission in Afghanistan over the period of this study is largely reassuring. Most personnel did not sustain mTBI while deployed, and most that did were free of PCS. As this research indicates, the strong association of PCS with mental health problems in this population warrants an approach where assessment of mental disorders should be a central focus of diagnosis and treatment for those with PCS.
